# Acute heart failure in non-cardiac surgery: A clinical consensus statement of the Association for Acute CardioVascular Care (ACVC) and the Heart Failure Association (HFA) of the ESC

**DOI:** 10.1093/eurheartj/ehaf559

**Published:** 2025-08-21

**Authors:** Danielle M Gualandro, Josep Masip, Sigrun Halvorsen, Susanna Price, Xavier Rosselló, Ovidiu Chioncel, W Frank Peacock, Òscar Miró, Mucio Tavares Oliveira Junior, Alexandre Mebazaa, Elke Platz, Offer Amir, Hannah Schaubroeck, Johannes Grand, Alessandro Sionis, Guido Tavazzi, Janine Pöss, Frederik H Verbrugge, Alessia Gambaro, Otilia Tica, Mattia Arrigo, Christian Mueller

**Affiliations:** Department of Cardiology and Cardiovascular Research Institute Basel (CRIB), University Hospital Basel, University of Basel, Spitalstrasse 2, Basel CH-4056, Switzerland; Consorci Sanitari Integral, University of Barcelona, Barcelona, Spain; Department of Cardiology, Oslo University Hospital Ulleval, Oslo, Norway; Department of Cardiology, University of Oslo, Oslo, Norway; Division of Heart, Lung and Critical Care, Royal Brompton Hospital, London, UK; Cardiology Department, Hospital Universitari Son Espases-IDISBA, Universitat de les Illes Baleares (UIB), Palma de Mallorca, Spain; Emergency Institute for Cardiovascular Diseases ‘Prof. C.C. Iliescu’, University of Medicine Carol Davila, Bucharest, Romania; Department of Emergency Medicine, Baylor College of Medicine, Houston, TX, USA; Emergency Department, Hospital Clínic, IDIBAPS, University of Barcelona, Barcelona, Catalonia, Spain; Heart Institute (InCor), University of Sao Paulo Medical School, Sao Paulo, Sao Paulo, Brazil; Department of Anesthesiology and Critical Care Unit, Université Paris Cité, APHP Hôpitaux Saint Louis & Lariboisière, Inserm 942 MASCOT, Paris, France; Cardiovascular Division, Brigham and Woman’s Hospital and Harvard Medical School, Boston, MA, USA; Heart Institute, Hadassah-Hebrew University Medical Center, Jerusalem, Israel; Department of Intensive Care Medicine, Internal Medicine and Pediatrics, Ghent University Hospital, Ghent, Belgium; Department of Cardiology, Copenhagen University Hospital Amager-Hvidovre, Hvidovre, Denmark; Department of Cardiology, Hospital de la Santa Creu I Sant Pau, Barcelona, Spain; Department of Clinical-surgical, Diagnostics and Pediatric Science, University of Pavia, Pavia, Italy; Department of Cardiology, Herzzentrum Leipzig, Leipzig, Germany; Centre for Cardiovascular Diseases, University Hospital Brussels, Laarbeeklaan 101, Jette 1090, Belgium; Faculty of Medicine and Pharmacy, Vrije Universiteit Brussel, Laarbeeklaan 101, Jette 1090, Belgium; Cardiology Division, Department of Medicine, Azienda Ospedaliera Universitaria Integrata Verona, Verona, Italy; Institute of Cardiovascular Sciences, University of Birmingham, Birmingham, UK; Cardiology Department, Emergency County Clinical Hospital of Bihor, Oradea, Romania; Department of Internal Medicine, Ente Ospedaliero Cantonale, Lugano, Switzerland; Faculty of Biomedical Sciences, Università della Svizzera Italiana, Lugano, Switzerland; Department of Cardiology and Cardiovascular Research Institute Basel (CRIB), University Hospital Basel, University of Basel, Spitalstrasse 2, Basel CH-4056, Switzerland

**Keywords:** Acute heart failure, Non-cardiac surgery, Perioperative complications, Heart failure, Natriuretic peptides

## Abstract

More than 64 million people worldwide have heart failure (HF), and these numbers are expected to rise. Acute HF (AHF) is the leading cause of hospitalization in patients over 65 years old and is linked to high mortality and readmission rates. AHF may also be a frequent complication in patients hospitalized for other medical reasons as well as after cardiac or non-cardiac surgery. These three entities are summarized as secondary AHF. As secondary AHF has been largely overlooked by medical research and education, little is known about its pathophysiology, phenotypes, diagnosis, management, and prognosis. Secondary AHF occurring after non-cardiac surgery warrants particular attention due to its very high mortality rates of up to 44% within 1 year and is therefore the focus of this review. The scope of this document is to summarize the available evidence regarding the pathophysiology, prevention, diagnosis, treatment, and prognosis of AHF after non-cardiac surgery. Key to prevention is understanding and addressing the pathophysiology of AHF after non-cardiac surgery, which involves close monitoring of fluid status to avoid volume overload and/or hypovolemia, avoiding hypo- and/or hypertension, treating pain and anaemia to prevent tachycardia, and avoiding electrolyte disturbances to prevent arrhythmias. Cardiac biomarkers, such as cardiac troponins and natriuretic peptides, serve as important diagnostic tools and enhance risk stratification in the perioperative setting. A low threshold to perform echocardiography in this population is suggested. Vigilant post-operative care is essential for the early recognition and treatment of AHF after non-cardiac surgery, which could help improve outcomes for patients.

## Graphical Abstract

**Graphical Abstract:**
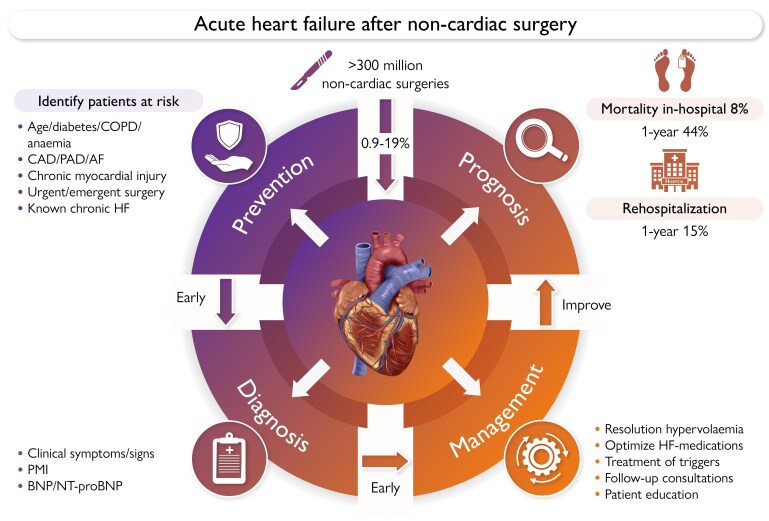
Prevention, diagnosis, management and prognosis of acute heart failure after non-cardiac surgery. HF, heart failure; COPD, chronic obstructive pulmonary disease; PAD, peripheral artery disease; CAD, coronary artery disease; AF, atrial fibrillation; PMI, perioperative myocardial infarction/injury; BNP, B-type natriuretic peptide; NT-proBNP, N-terminal pro-B-type natriuretic peptide.

## Introduction

Heart failure (HF) represents a global pandemic and is associated with high mortality and morbidity and significant healthcare costs. More than 64 million people worldwide have HF, and these numbers are expected to rise due to improvements in overall survival rates and better management of other underlying cardiac diseases, such as myocardial infarction (MI).^1,2^ Primary acute HF (AHF) is the leading cause of hospitalization in patients over 65 years old and is linked to high in-hospital (4%–10%) and 1-year (20%–30%) mortality rates, as well as high rates of rehospitalization.^3^

AHF may also be a frequent complication in patients hospitalized for other medical reasons, such as pneumonia, other infections, or after MI, as well as after cardiac or non-cardiac surgery.^4,5^ These three entities are summarized as secondary AHF. As secondary AHF has been largely overlooked by medical research and education, little is known about its pathophysiology, phenotypes, diagnosis, management, and prognosis. Secondary AHF occurring after non-cardiac surgery warrants particular attention for several reasons and is therefore the focus of this review.

First, AHF after non-cardiac surgery seems to be a major contributor to 30-day death after surgery.^6^ Considering that more than 300 million surgeries are performed worldwide each year,^7^ and an estimated 4.2 million of these patients die within 30 days, post-operative deaths are the third greatest contributor to global mortality, following ischaemic heart disease and stroke.^8^ Second, AHF after non-cardiac surgery is associated with very high mortality rates of up to 44% within 1 year.^9^ Third, these patients are mostly not seen by cardiologists. A better understanding of the current state of the art on AHF after non-cardiac surgery is necessary to mitigate the burden of this complication and improve prognosis after non-cardiac surgery.

The scope of this document is to summarize the available evidence and, in the absence of evidence, provide expert opinion regarding the pathophysiology, prevention, diagnosis, treatment, and prognosis of AHF after non-cardiac surgery.

## Incidence and prognosis of AHF after non-cardiac surgery

### Incidence

The precise incidence of AHF after non-cardiac surgery remains uncertain, primarily due to a scarcity of epidemiological studies that designate AHF as a primary endpoint. Over the past few decades, research on cardiac complications following non-cardiac surgery has primarily focused on cardiac ischaemic events, such as post-operative MI, and more recently, myocardial injury after non-cardiac surgery and perioperative myocardial infarction/injury (PMI). Traditionally, AHF after non-cardiac surgery has been included as part of a composite endpoint of major adverse cardiovascular events or recognized only when presenting as acute pulmonary oedema. Furthermore, some studies have concentrated on specific populations or surgical disciplines, and the diagnostic criteria used for AHF after non-cardiac surgery or even pulmonary oedema were not standardized. Given these limitations, reported incidences of AHF after non-cardiac surgery widely vary between 0.9% and 19%.^9–23^

A prospective multi-centre cohort study involving 3387 patients aged 40 years or older undergoing intermediate and high-risk non-cardiac surgery reported an incidence of AHF after non-cardiac surgery, diagnosed by the attending physician based on clinical symptoms and signs, of 1.2%,^16^ while a more recent prospective multi-centre cohort study of 9164 patients aged 65 years or older, or those over 45 years with diagnosed atherosclerotic disease undergoing major non-cardiac surgery (median age 73 years), found the incidence of AHF after non-cardiac surgery to be 2.5% in the overall population and 4.5% in the subgroup of those undergoing urgent or emergency surgeries.^9^ These data corroborate that older age is a major contributor to the incidence of AHF after non-cardiac surgery, similar to the incidence of HF outside the perioperative setting.^1^

Additionally, AHF after non-cardiac surgery appears to be more common following vascular, thoracic, and orthopaedic surgeries.^9,16,23^ It remains unclear whether this higher incidence is attributable to the particularities of these surgical procedures or the clinical characteristics of patients typically undergoing these procedures or the perioperative management of these patients.

### Prognosis

The prognosis of patients who experience AHF after non-cardiac surgery remains largely unknown, with most existing studies focusing on the outcomes of patients with known HF undergoing non-cardiac surgery. In a large cohort study including 21 560 996 non-cardiac surgeries, 4.9% of patients were identified as having perioperative HF. Any kind of perioperative HF was associated with higher in-hospital mortality [4.8% vs 0.78%, adjusted odds ratio (aOR) 2.2, 95% confidence interval (CI) 2.1–2.2]. Among the cases, in which it was possible to differentiate between acute and chronic HF, the in-hospital mortality of patients with AHF after non-cardiac surgery was 8%, higher than the mortality of patients with chronic HF (4%), and similar to the patients with acute-on-chronic HF (8%).^23^

Recently, a prospective cohort study including 9164 patients at increased cardiovascular risk undergoing major non-cardiac surgery, in which the main outcome of AHF after non-cardiac surgery was centrally adjudicated, revealed a very high 1-year mortality rate for these patients [44% vs 11% in patients without AHF after non-cardiac surgery, adjusted hazard ratio (aHR) 1.7, 95% CI, 1.3–2.2]. When further stratifying the cohort into four groups, patients with acute-on-chronic HF had the highest mortality (52%), followed by patients with *de novo* AHF after non-cardiac surgery (36%), patients with chronic HF without AHF (21%), and patients without HF (9%, *P* < .001).^9^ These findings highlight the higher mortality rates of AHF after non-cardiac surgery compared not only to primary AHF but also to secondary AHF outside the perioperative setting of non-cardiac surgery, which carries a 1-year mortality of approximately 20%.^5^

Furthermore, AHF after non-cardiac surgery was associated with a 15% risk of rehospitalization for AHF within 1 year.^9^

## Pathophysiology of AHF after non-cardiac surgery

The physiopathology of AHF after non-cardiac surgery is multi-factorial, involving complex interactions between the underlying structural heart disease (substrate), the trigger (in this case, the operation), and any amplifying mechanisms.^24^ The physiological and pathophysiological changes that occur in response to surgical trauma are influenced by the magnitude, invasiveness, type, and duration of the surgery. The surgical stress response encompasses both the neurohormonal and immune-inflammatory responses, but may also promote an imbalance in the coagulation system and can lead to the development of a systemic inflammatory response syndrome (SIRS), characterized by hypermetabolism and hypercatabolism.^25^

### Neurohormonal response

Surgical trauma activates the sympatho-adrenomedullary axis (SAM). Upon SAM activation, acetylcholine neurotransmitters are released, stimulating the adrenal glands to produce noradrenaline and adrenaline, resulting in increased blood pressure and heart rate. Blood flow is redirected towards vital organs, reducing supply to the kidneys and gastrointestinal system. This reduction activates the renin–angiotensin–aldosterone (RAA) system, affecting cardiovascular and renal systems, resulting in post-operative fluid retention, oliguria, increased blood volume, and elevated systemic vascular resistance.^25^ Glucagon secretion increases, prompting glycogenolysis and hepatic gluconeogenesis, while insulin secretion decreases, leading to a transient rise in post-operative blood glucose levels. There is also activation of the hypothalamic–pituitary–adrenal axis, with secretion of adrenocorticotropic hormone (ACTH) and vasopressin. ACTH promotes cortisol production from the adrenal cortex.^26^ Cortisol can remain elevated for up to 7 days after major surgery.^27^

### Immunoinflammatory response

Immediately after surgical trauma, the innate immune system is activated, and neutrophils and macrophages rapidly migrate to the injury site, phagocytizing damaged tissue and releasing cytokines, which are critical to the inflammatory response.^28^ Key pro-inflammatory cytokines include interleukin 1β (IL1β), interleukin 6 (IL6), interleukin 8 (IL8), and tumour necrosis factor-alpha (TNFα). Major anti-inflammatory cytokines include interleukin 4 (IL4), interleukin 10 (IL10), and IL1 receptor antagonist. Cytokines, particularly IL6, induce significant systemic changes, comprising the acute phase response, which increases liver proteins, including C-reactive protein (CRP), fibrinogen, and α2 macroglobulin, while albumin and transferrin levels decrease. CRP rises shortly after tissue injury and has a short half-life, making it a reliable post-operative biomarker of inflammation. Elevated CRP levels on the third to fourth post-operative day are linked to complications.^25^ Additionally, surgery provokes oxidative stress caused by reactive oxygen species generated from tissue injury and ischaemia–reperfusion lesions. On occasion, the immune system responds to surgical stress through an initial exaggerated inflammatory reaction leading to the SIRS.

### Coagulation system

Patients are at the highest risk of bleeding during surgery; however, these patients can develop hypercoagulability post-operatively as a result of changes in the plasma levels of coagulation factors and in platelet activation and impairment in post-operative fibrinolysis, which increases the likelihood of thrombotic events.^29,30^ Inflammatory processes characterized by high levels of inflammatory cytokines enhance coagulation by boosting the initiation phase, offering procoagulant surfaces for signal amplification and inhibiting natural anticoagulant mechanisms. Additionally, inflammatory mediators can elevate platelet count and increase their reactivity.^31^

### Anaesthesia

Anaesthetic medications and techniques can attenuate the surgical stress response and also impact the cardiovascular system. Volatile anaesthetics have reversible myocardial depressant effects and cause vascular smooth muscle relaxation, reducing systemic vascular resistance. Additionally, they suppress the release of cortisol and catecholamines. Intravenous anaesthetics agents, such as propofol, inhibit the sympathetic nervous system and exert anti-inflammatory and antioxidant effects, but also induce vasodilatation and hypotension. Opioids reduce cortisol secretion and have immunosuppressive effects. Regional anaesthesia (spinal/epidural) attenuates the neurohormonal response by reducing cortisol secretion and inducing sympathetic blockade, which lowers blood pressure and heart rate.^25^

### Development of AHF after non-cardiac surgery

AHF after non-cardiac surgery occurs when the heart is unable to meet the cardiac output required to match the systemic metabolic demands of the body and/or when there is impaired ventricular filling, leading to elevated cardiac filling pressures. It is postulated that the majority of AHF after non-cardiac surgery likely occurs in patients with decreased cardiovascular reserve prior to surgery, often due to underlying structural heart disease. Sympathetic nervous system stimulation, leading to tachycardia and hypertension, and impaired left ventricular (LV) relaxation increase LV and left atrial pressure, causing pulmonary congestion and triggering AHF after non-cardiac surgery. Intra-operative hypotension and bleeding can lead to vigorous fluid administration, further contributing to this process. Post-operatively, factors such as pain (resulting in catecholamine secretion, hypertension, and tachycardia), anaemia (demanding a higher cardiac output), cortisol, and activation of the RAA system (leading to sodium and water retention, increased blood volume, and elevated systemic vascular resistance) can exacerbate or perpetuate AHF after non-cardiac surgery. Oxidative stress, caused by reactive oxygen species generated during the perioperative period, can result in direct myocardial damage and accelerate HF progression. The occurrence of SIRS may lead to myocardial depression due to cytotoxic effects of pro-inflammatory cytokines. Myocardial injury can act both as a trigger and a consequence of AHF after non-cardiac surgery. Surgical stress (e.g. blood pressure fluctuations, tachycardia, anaemia, bleeding) can cause cardiac troponin (cTn) release due to myocardial oxygen supply–demand imbalance, leading to ischaemia. Conversely, acute cardiac volume or pressure overload in AHF after non-cardiac surgery can cause non-ischaemic cTn release secondary to myocardial stretch. Elevated circulating catecholamines, inflammatory cytokines, and oxidative stress may further contribute to cTn release. Impairment of LV relaxation may prolong compression of intra-myocardial arterioles, restricting early diastolic coronary flow, also contributing to imbalance in oxygen supply and demand.^32,33^ In patients with known HF with reduced LV ejection fraction (HFrEF), the interaction between the magnitude of these mechanisms and the severity of LV dysfunction may lead to cardiogenic shock. These patients could be classified according to the Society for Cardiovascular Angiography and Intervention (SCAI) as SCAI-A, e.g. patients at risk of developing cardiogenic shock. Closely monitoring these patients can lead to early recognition of cardiogenic shock allowing rapid initiation of appropriate interventions to reverse the underlying cause and the introduction of supportive therapies.^34^ Patients with known HF with preserved LVEF (HFpEF) have decreased venous capacitance and therefore increased vascular wall tension and are at increased risk of pulmonary oedema when exposed to surgical stress response and fluid overload.^35^ Cardiac arrhythmias are significant triggers for AHF after non-cardiac surgery, particularly atrial fibrillation (AF) with a high ventricular rate, in which the loss of left atrial contraction and reduced diastolic time lead to inadequate left ventricle filling and cardiac output.^36^

Unlike post-operative ischaemic events, which mostly occur within the first 2 days after surgery, about 50% AHF episodes occur within the first week after non-cardiac surgery, with a high risk of persisting up to 15 days and sometimes even 30 days post-operatively.^9^ Contributing factors for the occurrence of these late cases include failure to reinstitute HF therapy in known HF patients, unrecognized hypervolemic states post-surgery, delayed treatment in patients without an history of HF, and ongoing aggravating conditions such as anaemia, inflammation, fever, infections, and pain.

Less common mechanisms of AHF after non-cardiac surgery include Type 1 acute MI due to post-operative plaque rupture (caused by post-operative prothrombotic state),^37,38^ acute myocarditis, Takotsubo syndrome (more frequent up to 24 h after surgery),^39^ and acute right ventricular (RV) failure. Acute right HF can be caused by pulmonary embolism (thrombus, fat, bone cement, amniotic fluid, air/gas, etc.), acute respiratory distress syndrome (ARDS), RV MI, secondary to acute LV failure or decompensation of chronic RV failure, which can be due to primary RV dysfunction (RV cardiomyopathy, amyloidosis, cardiotoxicity, post-cardiac surgery, sarcoidosis, systemic sclerosis) or secondary RV dysfunction (pulmonary hypertension, tricuspid regurgitation, pulmonary regurgitation, etc.).^40–43^

The type of surgery is an important consideration in evaluating the mechanisms leading to AHF after non-cardiac surgery. For instance, after thoracic surgery, patients have a high incidence of AF^44,45^; after vascular surgery, patients are more likely to have coronary artery disease and ischaemic cardiopathy, predisposing them to ischaemic events and AHF after non-cardiac surgery^23,46^; and after major orthopaedic surgery, patients often have known or unrecognized HFpEF, making them more susceptible to AHF after non-cardiac surgery due to volume overload, tachycardia, and bleeding.^23^

**Figure 1:**
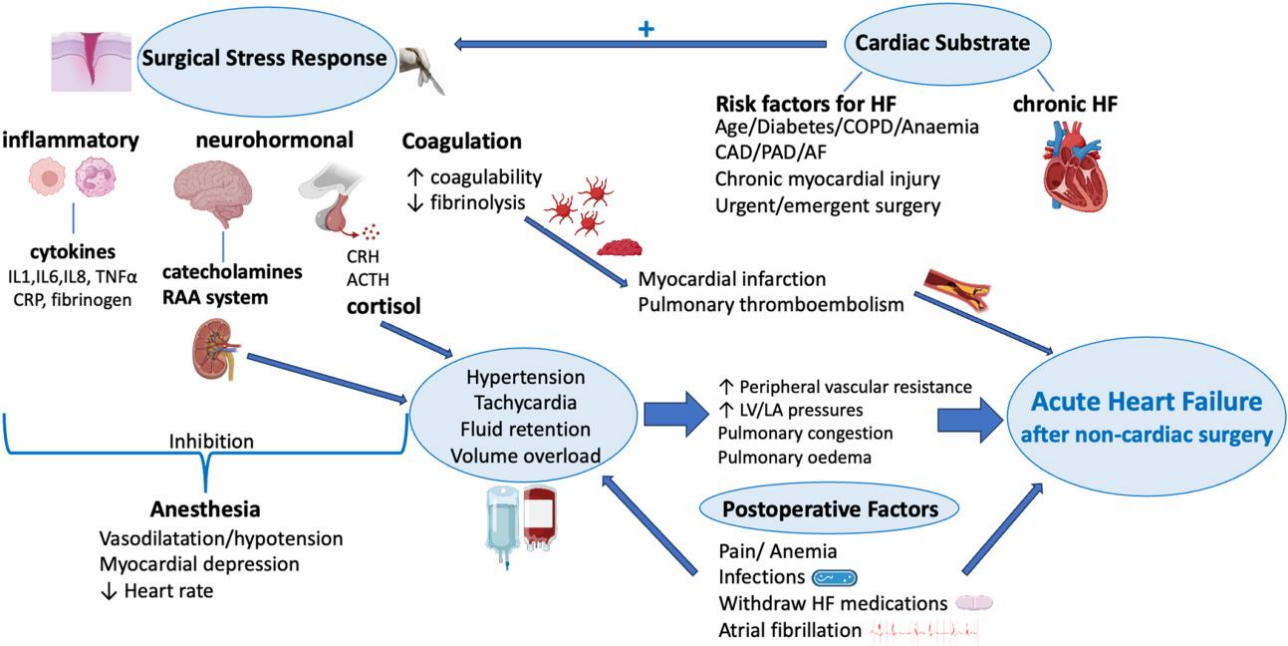
Pathophysiology of acute heart failure after non-cardiac surgery. CRH, corticotropin-releasing hormone; ACTH, adrenocorticotropic hormone; IL, interleukin; TNFα:, tumour necrosis factor-alpha; CRP, C-reactive protein; RAA, renin–angiotensin–aldosterone; HF, heart failure; COPD, chronic obstructive pulmonary disease; PAD, peripheral artery disease; CAD, coronary artery disease; AF, atrial fibrillation; LV, left ventricle; LA, left atrium

In summary, the pathophysiology of AHF after non-cardiac surgery is multi-factorial, due to the interaction between the substrate (underlying heart disease), the magnitude of the surgical stress, the effects of anaesthesia, intra-operative haemodynamic and volume management, and post-operative amplifying factors and their management (*Figure 1*).

## Pre-operative risk assessment and management

### Patients with known HF

The prevalence of chronic HF in patients undergoing non-cardiac surgery varies between 3% and 12%, depending on the population studied.^9,47,48^ In a large contemporary Swedish cohort including 283 632 patients, the prevalence of pre-operative HF was 3.6% in elective surgeries and 6.6% in emergency surgeries. Among patients older than 65 years of age, the prevalence increases to 6.6% in elective surgeries and 12.5% in emergency surgeries.^49^ Due to the retrospective nature of most studies, the true prevalence of pre-operative HF may have been underestimated and remains to be confirmed in prospective studies focusing specifically on HF.

#### Perioperative risk in patients with HF

Chronic HF is a known risk factor for mortality after non-cardiac surgery and is therefore included in commonly used risk scores.^50–57^ In the recent Swedish cohort study of 283 632 patients, mortality rates for HF patients undergoing elective surgery were 3% at 30 days, 7% at 90 days, and 16% at 1 year—over five times higher than patients without HF. After adjusting for confounders, the mortality risk remained 80% higher in HF patients. For emergency surgery, mortality rates were 14% at 30 days, 22% at 90 , and 39% at 1 year.^49^

A study from the US Veterans Affairs Surgical Quality Improvement Project evaluated 609 735 patients undergoing non-cardiac surgery, of whom 7.9% had HF pre-operatively. Patients were classified based on LVEF and symptom presence. Sixty per cent had LVEF >50%, 16% had LVEF 40%–49%, and 22% had LVEF <40%, while 12% presented with HF symptoms. Adjusted analyses showed that HF presence significantly increased 90 day mortality, regardless of symptoms or LVEF, though symptomatic patients had a higher mortality risk. Lower LVEF was associated with higher mortality, with those having LVEF <30% facing the worst prognosis.^48^

Pre-operative HF not only increases the risk of perioperative death but also AHF after non-cardiac surgery (10%–25%).^9,58,59^

#### Risk stratification and pre-operative management of patients with HF

The performance of commonly used risk scores in the specific populations of HF patients is largely unknown.^51–53,60,61^ Therefore, a risk score specific for HF patients was developed in 16 827 HF patients (Andersson's score), of whom 1787 (10.6%) died within 30 days post-surgery. The *c*-statistic was 0.79, and calibration was reasonable. Mortality risk ranged from <2% for a score <5 to >50% for a score ≥20 (*Table 1*).^62^ This score could be useful for stratifying mortality risk in HF patients undergoing non-cardiac surgery, but it still requires external validation.

**Table 1:** Risk stratification scores

	Revised cardiac risk index		Andersson's score	
Population	General population		Patients with known HF	
Variables		Points		Points
	Ischaemic heart disease	1	Male sex	1
	Cerebrovascular disease	1	Cerebrovascular disease	1
	History of HF	1	Diabetes with insulin	1
	Diabetes with insulin	1	Renal disease	1
	Serum creatinine level ≥2 mg/dL	1	Age 55–65 years	2
	High-risk surgery^a^	1	Age 66–75 years	4
			Age 75–85 years	5
			Age >85 years	7
			BMI < 18.5 kg/m^2^	4
			BMI 18.5–25 kg/m^2^	3
			BMI 25–30 kg/m^2^	1
			BMI >30 kg/m^2^	0
			Emergency surgery	5
			High-risk surgery	3
Derivation population	1422 patients		16 827 HF patients	
Outcomes	MI, death, cardiac arrest in 30 days		Mortality	
Outcome rates	Class I (0 points)	3.9%	<5 points	<2%
	Class II (1 point)	6%	5–8 points	2%–5%
	Class III (2 points)	10%	9–11 points	5%–10%
	Class IV (≥3 points)	15%	12–14 points	10%–20%
			15–16 points	20%–30%
			17–19 points	30%–40%
			>20 points	>50%

HF, heart failure; BMI, body mass index; MI, myocardial infarction.^51,62^

^a^Intra-thoracic, intra-abdominal, or vascular supra-inguinal.

The current 2022 ESC Guidelines on cardiovascular assessment and management of patients undergoing non-cardiac surgery^54^ recommends that, during the pre-operative consultation, symptoms according to the New York Heart Association (NYHA) class, signs of HF, current medications, LVEF, high-sensitivity cTn (hs-cTn), and natriuretic peptides (NPs) must be assessed. In stable patients who had an echocardiogram in the last 6 months, a repeat echocardiogram seemed redundant.^3,54,63^ It is recommended by the same ESC Guidelines that patients with HF undergoing non-cardiac surgery receive optimal medical treatment.^54^ This, of course, particularly applies to patients with known HF (Stages C and D according to the 2022 AHA/ACC/HFSA Heart failure guidelines).^64^ Special attention should be given to the daily monitoring of fluid balance, since high-volume and high-sodium infusions are often administered in the perioperative period.

**Figure 2:**
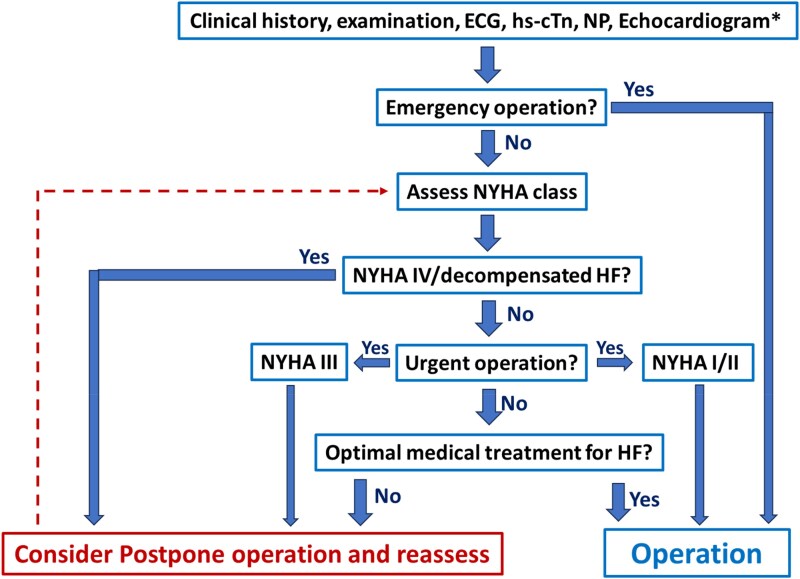
Proposed algorithm for pre-operative risk assessment in patients with known HF. *If not performed in the last 6 months. ECG, electrocardiogram; hs-cTn, high-sensitivity cardiac troponin; NP, natriuretic peptides; NYHA, New York Heart Association; HF, heart failure

For patients with clinical signs of AHF (e.g. pulmonary rales, elevated jugular vein distension, peripheral oedema, third heart sound, cold extremities), NYHA Class IV, or HF Stage D, surgery should be postponed until clinical compensation is achieved, except in cases where emergency surgery is required (*Figure 2*). In patients with NYHA Class III symptoms, postponing surgery until clinical improvement, decongestion (in case of urgent or time-sensitive surgery), and up-titration of HF medications (in case of elective surgery) is reasonable. All patients with known HF, including those with NYHA Class I/II (Stages B/C), should be on maximally tolerated guideline-recommended optimal medical therapy before elective surgery.^54,57^

In emergency surgery, standard pre-operative optimization is not feasible, but post-operative management should be planned and recommended. The pre- and intra-operative optimization remain at the discretion of the anaesthesiologists. For urgent surgery, if the patient shows clinical signs of decompensation, introducing intravenous diuretics for rapid resolution of congestion is reasonable and may prevent further decompensation after surgery. Managing patients undergoing ‘time-sensitive’ surgeries, such as oncological procedures, is challenging. In these cases, weighing the risk of post-operative mortality against the risk of disease progression by delaying surgery requires careful consideration. A patient-centred management driven by a multi-disciplinary approach involving all specialists in a pre-operative meeting seems advisable.

In principle, all specific HF medications should be continued during the perioperative period.^54^ However, there have been concerns regarding the risk of refractory hypotension associated with continuation of renin–angiotensin system inhibitors (RASI) before non-cardiac surgery, but recent randomized studies, including the STOP-or-NOT trial, demonstrated that a continuation strategy of RASI before surgery was not associated with a higher rate of post-operative complications than a discontinuation strategy in a general population of patients undergoing non-cardiac surgery.^65,66^ There is controversy regarding the possible need to discontinue SGLT2 inhibitors due to the small risk of euglycemic ketoacidosis in patients with diabetes mellitus.^54^

The ongoing PeriOP-CARE HF trial, a randomized controlled trial designed to evaluate if a standardized approach to the pre-operative evaluation, optimization, and perioperative management will reduce post-operative morbidity in HF patients aged 65 years or older undergoing non-cardiac surgery with intermediate or high surgical risk, may provide evidence-based approaches to these patients in the future.^67^

A particular situation is the perioperative management of patients with advanced HF supported with LV assist devices (LVADs). Small studies have suggested that it is feasible to perform non-cardiac surgery in these patients, although the perioperative management of these patients is complex, due to the perioperative risk of bleeding and thrombotic complications, the risk of injury or contamination of the VAD driveline, and challenges in haemodynamic monitoring and management.^68^ Therefore, all LVAD patients undergoing non-cardiac surgery should have a multi-disciplinary team of specialists, including surgeons (cardiac and non-cardiac), anaesthesiologists, HF cardiologists, and dedicated VAD personnel co-ordinating their care.^69^ The perioperative approach to patients with VAD undergoing non-cardiac surgery is described in *Table 2*.^54^

**Table 2:** Perioperative management of patients with ventricular assist devices undergoing non-cardiac surgery

Pre-operative	Intra-operative	Post-operative
Multi-disciplinary team identified (primary surgical and anaesthesia teams, cardiac surgery, HF cardiologist, VAD personnel) Pre-operative medical optimization when possible or necessary Physical examination focused on the sequelae of HF Baseline ECG, echocardiogram, and laboratory values Manage pacemaker/ICD settings when indicated CT examination to evaluate possible driveline interference with the operative field Hold, bridge, or reverse anticoagulation when indicated, after VAD team consultation	Standard American Society of Anesthesiologists monitors Cerebral tissue oxygenation, processed electroencephalogram, arterial line with ultrasound guidance, central venous catheter if fluid shifts are expected, PA catheter only if severe pulmonary hypertension, TEE available Monitor VAD control console External defibrillator pads in place Optimize pre-load, support RV function, avoid increase in afterload Gradual peritoneal insufflations and position changes	Standard post-anaesthesia care unit unless ICU is otherwise indicated Extubation criteria are unchanged Avoid hypoventilation, optimize oxygenation Resume heparin infusion when post-op bleeding risk is acceptable

Adapted from Halvorsen *et al*.^54^

HF, heart failure; VAD, ventricular assist device; ICD, Implantable cardioverter–defibrillator; ECG, electrocardiogram; CT, computed tomography; TEE, transoesophageal echocardiography; ICU, intensive care unit; PA, pulmonary artery; RV, right ventricular.

In summary, perioperative management of patients with HF include close clinical and haemodynamic monitoring, serial volume status assessments, maintenance of guideline-directed medical recommended therapy, and their reintroduction as early as possible post-operatively.

### Patients without known HF

Identifying high-risk patients through comprehensive pre-operative assessment is key, considering that nearly half of the patients developing AHF after non-cardiac surgery do not have known HF (*de novo* AHF after non-cardiac surgery). Very often, their baseline characteristics may allow categorization as Stage A or Stage B HF, according to the universal definition of HF.^70^ It may be also possible that undetected congestion, at the time of hospital admission, may contribute to erroneous classification and missed HF diagnosis. Independent predictors of AHF after non-cardiac surgery include age, coronary artery disease, peripheral artery disease, diabetes, urgent/emergent surgery, chronic HF, AF, chronic obstructive pulmonary disease (COPD), anaemia, and chronic myocardial injury.^9,71^

Moreover, undiagnosed HF seems to be a problem in elderly patients undergoing major non-cardiac surgery. A recent study demonstrated that only about half of patients with centrally adjudicated HF pre-operatively had been identified in routine clinical care.^72^ Therefore, during pre-operative evaluation, high awareness for HF combined possibly with active surveillance using B-type NP (BNP) or N-terminal pro-B-type NP (NT-proBNP) testing (*Table 3*) will help in the detection of previously undiagnosed HF.^3,73,74^ Special attention should be given to actively questioning at-risk patients about HF symptoms. As HF is an insidious disease, elderly patients may attribute their effort intolerance or inability to climb stairs to their normal aging, which might actually be HF manifestations.

**Table 3:** Natriuretic peptides cut-offs for diagnose of chronic and AHF

Chronic HF	NT-proBNP (ng/L)	BNP (ng/L)
HF unlikely	<125	<35
HF likely	≥300 (SR)	≥80 (SR)
	≥600 (AF)	≥150 (AF)

AHF	Age <50 years	NT-proBNP (ng/L) Age 50–75 years	Age >75 years	BNP (ng/L)
HF unlikely	<300	<300	<300	<100
HF likely	>450	>900	>1800	>400

In patients with BMI ≥ 35kg/m^2^, cut-offs have to be reduced by 50%.

HF, heart failure; NT-pro BNP, N-terminal pro-B-type natriuretic peptide; BNP, B-type natriuretic peptide; BMI, body mass index; SR, sinus rhythm; AF, atrial fibrillation.^63,73–76^

#### Patients with dyspnoea

For patients with dyspnoea and/or leg oedema, irrespective of the planned surgery, clinical assessment, an electrocardiogram (ECG), and serum levels of BNP or NT-proBNP help in the assessment to determine whether HF is the most likely cause.^3,73,75,76^ Clinical suspicion, elevated NPs (*Table 3*), or heart murmurs warrant further testing with an echocardiogram.^54^ If HF is diagnosed, risk assessment and pre-operative management should follow the steps described in the ‘Patients with known HF’ section and *Figure 2*.

#### Asymptomatic patients

**Figure 3:**
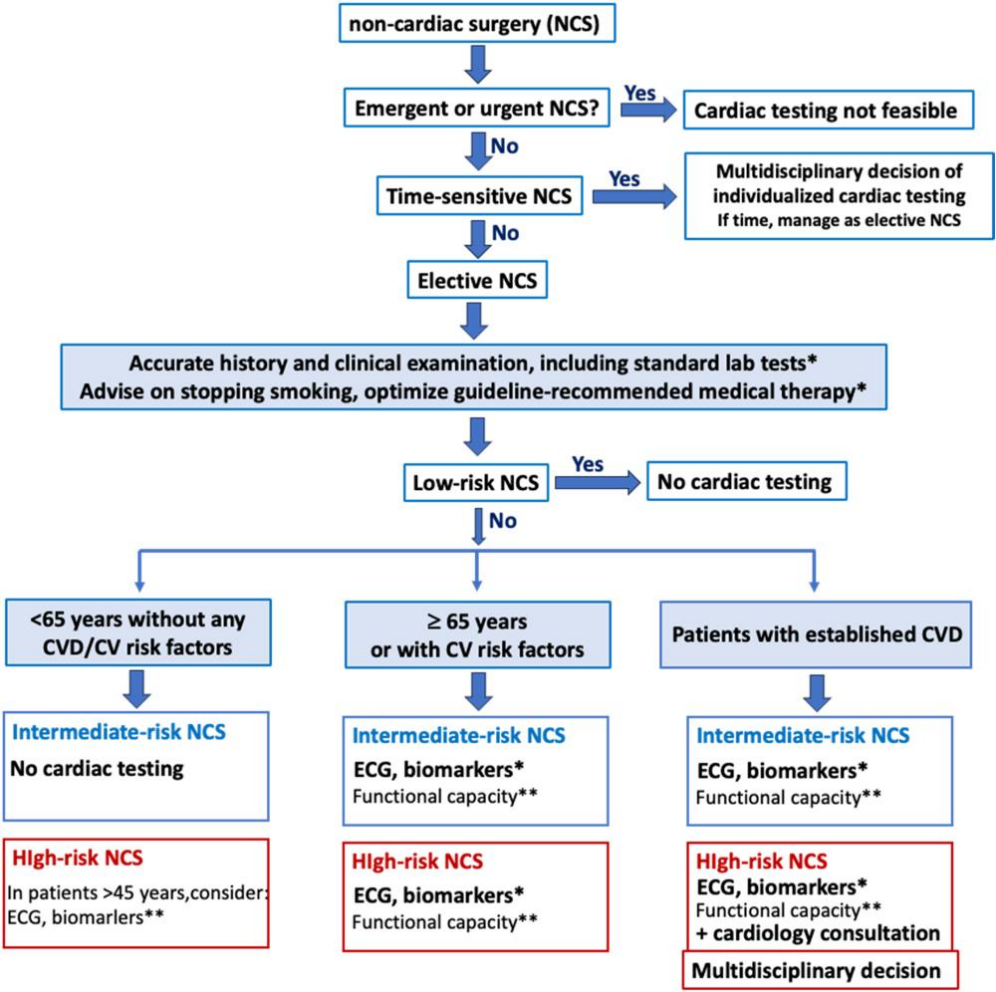
Pre-operative risk stratification in general. Modified from Halvorsen *et al*.^54^ CV, cardiovascular; CVD, cardiovascular disease; ECG, electrocardiogram; NCS, non-cardiac surgery. *Class I recommendation according to the 2022 ESC Guidelines on cardiovascular assessment and management of patients undergoing non-cardiac surgery. **Class IIa recommendation according to the 2022 ESC Guidelines on cardiovascular assessment and management of patients undergoing non-cardiac surgery. CV risk factors: hypertension, smoking, dyslipidemia, diabetes, family history of CVD. Biomarkers: hs-cTn T/I and/ or BNP/NT-proBNP. Functional capacity based on Duke Activity Status Index (DASI) or the ability to climb two flights of stairs

In most asymptomatic patients scheduled for non-cardiac surgery, detailed clinical assessment without additional investigations is sufficient. However, the 12-lead ECG and hs-cTn can complement clinical assessment in patients above the age of 65 years or in those with known cardiovascular disease or cardiovascular risk factors undergoing intermediate or high-risk surgery (*Figure 3*).^54^

Perioperative screening with hs-cTn can help identify patients with pre-operative chronic HF, as HF often causes chronic myocardial injury (i.e. chronic elevation of hs-cTn above the 99th percentile of the upper reference limit).^71^ Elevated pre-operative hs-cTn concentrations are associated with increased post-operative mortality and cardiac complications.^10,77–79^ Recently, chronic myocardial injury has also been identified as an independent predictor of AHF after non-cardiac surgery.^9^ BNP/NT-proBNP testing and an echocardiogram seems reasonable in patients with elevated hs-cTn concentrations.

## Surveillance and diagnosis of AHF after non-cardiac surgery

The diagnosis of AHF after non-cardiac surgery can be made in two ways. The traditional way occurs in patients developing severe enough acute dyspnoea after surgery, triggering detailed clinical assessment and work-up, which then subsequently lead to the identification of AHF as the cause of symptoms. However, specific challenges apply to detecting post-operative cardiac complications in general and AHF after non-cardiac surgery in particular. First, due to anaesthesia and opioids, symptoms of AHF after non-cardiac surgery may be less severe or atypical including abdominal discomfort, nausea, and vomiting caused by hepatic/gastrointestinal congestion due to right-sided overload. Additionally, AHF after non-cardiac surgery could be missed by attributing its symptoms to other aspects including post-operative nausea and vomiting, post-surgical fatigue, post-operative pain, and drains. Second, cardiologists are usually not directly involved in post-operative care; therefore, the early detection and treatment of AHF after non-cardiac surgery is performed by non-cardiologists, sometimes with little training in the early detection of acute cardiac disorders. Additionally, RV dysfunction could be an overlooked cause of immediate post-operative haemodynamic deterioration, particularly in patients under mechanical ventilation, patients after thoracic surgery, and in the presence of ARDS.^40,43,80,81^

**Figure 4:**
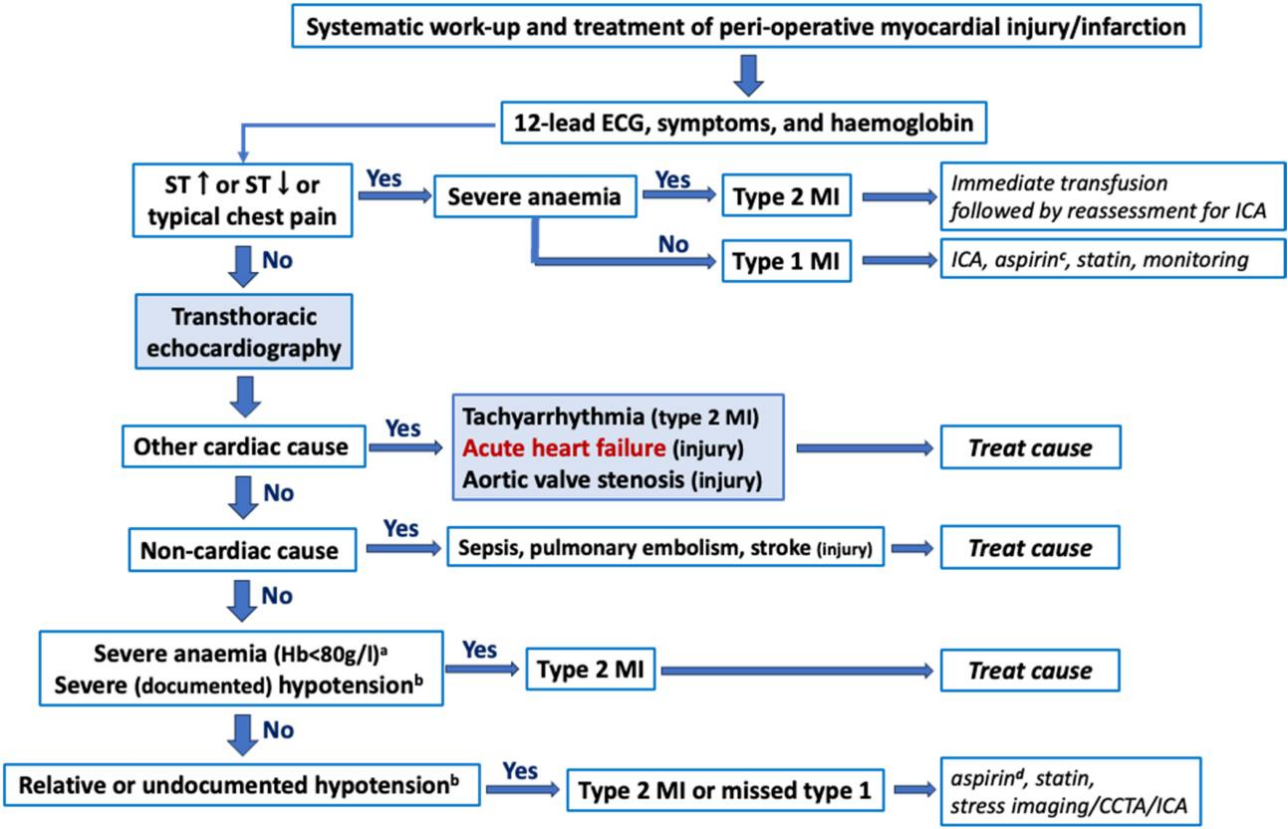
Perioperative myocardial infarction/injury systematic work-up. Modified From Halvorsen *et al*.^54^ ECG, electrocardiogram; ST, ST-segment; MI, myocardial infarction; ICA, invasive coronary angiography; Hb, haemoglobin; CCTA, coronary computed tomography angiography. ^a^Or active bleeding. ^b^Other Type 2 MI trigger such as hypoxaemia, tachycardia, and hypertension. ^c^Dual antiplatelet therapy after coronary stenting. ^d^Possibly in combination with dabigatran 110 mg b.i.d. Most patients with Type 2 MI and silent Type 1 MI should be scheduled for stress imaging or CCTA/ICA as outpatients after discharge, depending on symptoms prior to or after surgery and known CAD

The second way for diagnosing AHF after non-cardiac surgery is during active surveillance for PMI, as recommended by the 2022 ESC Guidelines.^54^ When a PMI (defined as an absolute delta of the upper reference limit (URL) of the hs-cTn assay above pre-operative concentrations or between two post-operative concentrations if the pre-operative value was missing) is detected, a work-up is triggered that then identifies AHF after non-cardiac surgery as the cause of PMI (*Figure 4*).^54,82^

High awareness for AHF after non-cardiac surgery, detailed clinical assessment, and BNP or NT-proBNP testing are mandatory not to miss AHF after non-cardiac surgery. The cut-off levels of NPs used for diagnosing AHF in patients presenting at the emergency department (*Table 3*) have not been validated for the post-operative period, but they can offer some useful guidance. It has been shown in an individual patient data meta-analysis of 2167 patients with both pre- and post-operative BNP/NT-proBNP measurements that after surgery, NP concentrations increased in 76% of patients [median BNP increase of 66 ng/L, interquartile range (IQR) 123 ng/L and median NT-proBNP 323 ng/L, IQR 874 ng/L].^83^ In a cohort of 2051 patients who had NP measured within 7 days post-surgery, a post-operative BNP >245 ng/L and NT-proBNP >718 ng/L independently predicted HF within 30 days. Unfortunately, these thresholds were developed using receiver operating curves for prediction of combined endpoint of MI and mortality, and not AHF after non-cardiac surgery.^84^

It is crucial to consider other causes of dyspnoea/hypoxemia post-surgery, such as pneumonia, atelectasis, pulmonary embolism, COPD, and metabolic disorders. These conditions could either trigger or coexist with AHF after non-cardiac surgery and should always be considered in differential diagnosis.

## Management of AHF after non-cardiac surgery

**Figure 5:**
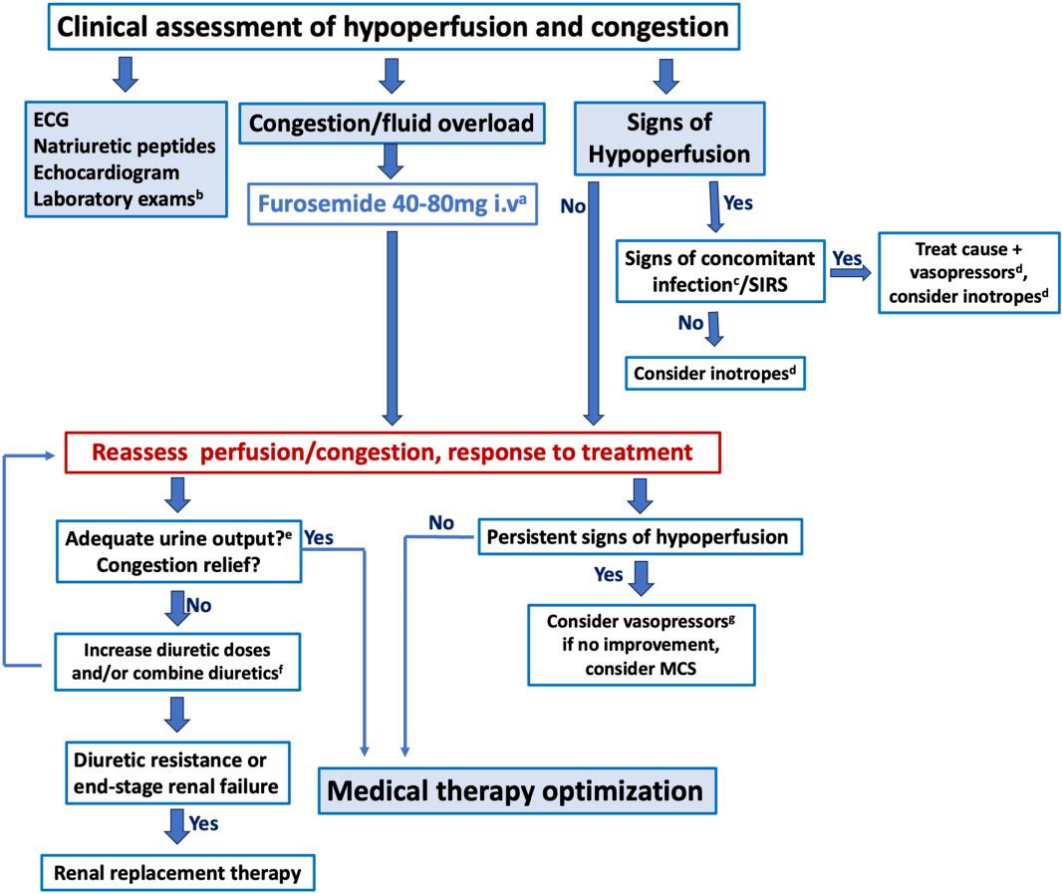
Management of patients with AHF after non-cardiac surgery. Modified from McDonagh TA *et al*.^3^ ECG, electrocardiogram; SIRS, systemic inflammatory response syndrome; MCS, mechanical cardiac support. ^a^1 mg/kg or 50% of the total daily oral dose previously administered. ^b^Initial exams: troponin, serum creatinine, electrolytes, blood urea nitrogen or urea, haemoglobin. ^c^Sampling, liberal imaging to search for infect, as well as liberal introduction of antibiotics. ^d^Additional arguments for inotropes vs vasopressors: low diastolic blood pressure = vasopressors preferred; proportional pulse pressure ≤25% = inotropes preferred. ^e^Urine output >100–150 mL/h during the first 6 h; if monitoring not possible, at least 2–3 times diuresis within 2 h. ^f^Acetazolamide 500 mg i.v. or p.o. for 3–5 days OR metolazone 5 mg p.o. for 3–5 days. ^g^Preferable norepinephrine.

The treatment of AHF after non-cardiac surgery is in line with the management of primary AHF as outlined in current guidelines.^3,63^ However, specific considerations pertinent to the perioperative setting have to be considered. A flowchart for the management of AHF after non-cardiac surgery is shown in *Figure 5*.

The primary goal of therapy is to resolve congestion. Intravenous loop diuretics serve as the cornerstone of initial management. A bolus dose of furosemide 1 mg/kg or at least 40 mg intravenously, or 50% of the total daily oral dose previously administered, should be given, followed by response assessment and dose adjustments. Close monitoring of electrolytes is essential. Mineralocorticoid receptor antagonists (MRAs) such as spironolactone or eplerenone can be initiated early to prevent hypokalaemia induced by loop diuretics. If there is insufficient response to initial therapy, additional diuretics acting at different sites, such as thiazides, metolazone, or acetazolamide, may be considered (*Table 4*).^3^ Patients submitted to surgeries with high volume loading deserve special attention regarding clinical monitoring of signs of volume overload and prompt initiation of diuretics.^88^

**Table 4:** Drugs used to treat AHF

Diuretics		
Therapeutic goals	Urine output >100–150 mL/h/first 6 h Achieve complete decongestion	

Drug	Dose	Pharmacology
Furosemide	Bolus 1 mg/kg or 40–80 mg i.v. or 50% previous daily doses 2–4 daily boluses Maximal 1000 mg/day	Class: *loop diuretics* Site of action: *ascending loop of Henle* Onset: *p.o.: 0.5–1* *h, i.v.: 5–10* *min;* Half-life: *1.5–3.0* *h*
Torasemide	Bolus 10–20 mg i.v. Maximal 200–300 mg/day	Class: *loop diuretics* Site of action: *ascending loop of Henle* Onset: *p.o.: 0.5–1* *h, i.v.: 5–10* *min* Half-life: *3–6* *h*
Acetazolamide	500 mg i.v. or p.o. *o.d*. for 3–5 days	Site of action: *proximal nephron* Onset: *p.o.: 1* *h, i.v.: 15–60* *min* half-life: *2.4–5.4* *h*
Metolazone	2.5–10 mg p.o. *o.d*.	Class: *thiazide-like diuretics* Site of action: *early distal convoluted tubule* Onset: *p.o.: 1–2.5* *h* Half-life: *6–20* *h*
Hydrochlorothiazide	12.5–100 mg p.o. *o.d*.	Class: *thiazide-like diuretics* Site of action: *early distal convoluted tubule* Onset: *p.o.: 1–2.5* *h* Half-life: *6–15* *h*
Spironolactone	25–50 mg p.o. *o.d*.	Class: *MRA* Site of action: *late distal tubule* Onset: *p.o.: 48–72 h* Half-life: *16.5 h*
Eplerenone	25–50 mg p.o. *o.d*.	Class: *MRA* Site of action: *late distal tubule* Onset: *p.o.: 48–72 h* Half-life: *3–6 h*

Intravenous vasodilators		
Therapeutic goals	Improvement of lung congestion and symptoms, improve forward stroke volume in low cardiac output with preserved blood pressure, and decrease blood pressure in hypertension	

Drug	Dose	Pharmacology
Nitroglycerine	Start: 10–20 µg/min, increase up to 200 µg/min	*Converts to NO, activates guanylate cyclase → ↑ cGMP → smooth muscle relaxation → vasodilation (predominantly venous dilation at lower doses, arterial dilation at higher doses)* Onset: *ca. 2 min* Half-life: ca. *1–3 min.*
Nitroprusside	Start: 0.3 µg/kg/min, increase up to 5 µg/kg/min	*Releases NO directly → activates guanylate cyclase → ↑ cGMP → smooth muscle relaxation → balanced arterial and venous vasodilation*. Onset: *<1* *min* Half-life: *< 2* *min*
Isosorbide dinitrate	Start: 1 mg/h, increase up to 10 mg/h	*Metabolized to NO, activates guanylate cyclase → ↑ cGMP → smooth muscle relaxation → vasodilation (primarily venous dilation)* Onset: *1–2* *min* Half-life: ca. *9–10* *min*

Inotropes/vasopressors		
Therapeutic goals	Improvement of organ perfusion	

Drug	Dose	Pharmacology
Dobutamine	2–20 µg/kg/min	Class: *β-agonist* *β1-receptor agonist, ↑ adenylate cyclase activity, ↑cAMP, ↑ calcium influx, ↑ contractility* Onset: *1–2* *min* Half-life: *2* *min*
Milrinone	0.375–0.75 µg/kg/min	Class: *phosphodiesterase inhibitors* *Inhibit phosphodiesterase III, ↑ cAMP, ↑ calcium influx, ↑ contractility, vasodilatation* Onset: *5–15* *min* Half-life: ca. *2* *h*
Levosimendan	0.05–2.0 µg/kg/min^a^	Class: *calcium sensitizers* *↑ sensitivity of cardiac troponin C to calcium, ↑ contractility* *opens ATP-sensitive potassium channels* → *vasodilatation* Onset: 1 h Half-life: *75–80* *h (active metabolite OR-1896)*
Norepinephrine	0.02–1.0 µg/kg/min	Class: *α₁, α₂, and β₁-adrenergic agonist* *α₁-adrenergic receptors → vasoconstriction → ↑SVR, ↑BP* *β₁-adrenergic stimulation → ↑myocardial contractility and HR* Onset: *<2 min* Half-life: ca. *2 min.*

MRA, mineralocorticoid receptor antagonist; i.v., intravenous; p.o., *per os*; o.d., once daily; min, minutes; h, hours.

^a^Usually 0.1 µg/kg/min; NO, nitric oxide; SVR, systemic vascular resistance; BP, blood pressure; HR, heart rate; cAMP, cyclic AMP.^3,85–87^.

Patients with pulmonary oedema and systolic blood pressure ≥110 mmHg may benefit from intravenous or transdermal nitrates and should receive non-invasive positive pressure ventilation as soon as possible to reduce symptom worsening and the need for endotracheal intubation, unless contra-indicated (*Table 4*). Early detection and management of complications, such as arrhythmias and low output syndrome, are critical for optimal outcomes. Applying the SCAI classification in advance seems reasonable to promptly recognize cardiogenic shock.^34^ Inotropes (*Table 4*) are an option for patients with hypoperfusion.^3^

Patients with isolated RV failure deserve specific considerations. The right ventricle is volume-tolerant but pressure intolerant. Once RV failure is identified, measures should be taken to avoid the installation of a vicious circle of systemic hypotension with RV ischaemia and dilatation, which can lead to rapid haemodynamic decline. The most important intervention is to correct hypotension through the optimization of volume status and administration of vasopressors. Volemia must receive particular attention because patients with RV dysfunction are pre-load dependent, but also tolerate hypervolemia poorly, developing RV dilatation and failure. Potential causes of increase pulmonary vascular resistance, such as hypoxia and hypercarbia, must be corrected, keeping in mind that in positive pressure ventilation, inspiration leads to a general increase in RV afterload and drop in RV pre-load. If there is persistent RV failure, selective pulmonary vasodilatation with inhaled nitric oxide or prostaglandins should be considered. The use of inotropes may be necessary to maintain cardiac output and systemic perfusion. Determination and treatment of the underlying cause is essential. Perioperative RV failure is most often, although not exclusively, secondary to acute pulmonary hypertension (increased afterload), and pulmonary embolism should be proactively investigated.^40,42,43^

Following echocardiography and determination of LVEF, the main medication classes recommended by current HF guidelines should be progressively introduced before discharge. These include angiotensin-converting enzyme inhibitor (ACE-I) or angiotensin receptor-neprilysin inhibitors (ARNI), MRAs, beta-blockers, and SGLT2 inhibitors for HFrEF, and SGLT2 inhibitors for HFpEF.^3,63^ A recent randomized controlled trial showed that finerenone in patients with HF with mildly reduced LVEF (HFmrEF) and HFpEF reduced rate of a composite of total worsening HF events and death from cardiovascular causes than placebo.^89^ Additional patient-level meta-analysis of randomized trials suggested that the use of MRAs in HFmrEF and HFpEF reduces the risk of re-hospitalizations.^90^ Although this evidence has not yet been incorporated into ESC Guidelines, it seems reasonable to start an MRA in patients with HFmrEF and HFpEF. It is important to note that caution is needed with the introduction and up-titration of beta-blockers in patients with HFrEF and an acute decompensation. If specific causes of AHF after non-cardiac surgery, such as severe valve disease, MI, or pulmonary embolism, are identified, management should follow current guidelines.^38,91,92^

Patients in the post-operative period may present with comorbidities or non-cardiac complications requiring medications that could interact with HF management. Many intravenous medications are routinely diluted in large volumes of saline, potentially exacerbating hypervolemia. Interdisciplinary collaboration, including nursing staff, is crucial to minimize the fluids administration, optimizing treatment efficacy. Additionally, the administration of blood products can contribute to volume overload, and an extra dose of intravenous loop diuretics may help mitigate this issue. Finally, it is advisable to check for interactions between the medicaments used to treat HF and other medications commonly used after surgery, such as antibiotics or antifungal agents and proton pump inhibitors. For example, amphotericin B, an antifungal agent, is associated with the incidence of hypokalaemia especially when used concomitantly with loop diuretics. Furosemide has been shown to increase the plasma concentrations and/or reduce the clearance of several cephalosporins. Ototoxicity-associated with aminoglycoside antibiotics is potentiated by also by the coadministration of a loop diuretic. Loop diuretics can also lead to hypomagnesemia when associated with proton pump inhibitors, such as esomeprazole, lansoprazole, and omeprazole.^93^ In patients receiving ACE-I/ARB/ARNI, the use of trimethoprim, pentamidine, or fluconazole could cause hyperkalaemia. Antifungal drugs, such as fluconazole and ketoconazole, inhibit the metabolism of losartan.^94^

As SGLT2 inhibitors are currently indicated in the treatment of HF regardless of the LVEF, some particularities of these medications have to be highlighted. These medications are contra-indicated in patients with Type 1 diabetes due to the risk of euglycemic ketoacidosis. However, it has been shown that the euglycemic ketoacidosis can also happen in patients with Type 2 diabetes in the presence of certain risk triggers, such as prolonged fasting, infection, trauma, and surgery. The usual symptoms are fatigue, nausea, vomiting, dyspnoea, abdominal pain, altered mental status, and seizures.^95^ As the symptoms are unspecific and frequently observed due to other causes after surgery, the diagnosis of ketoacidosis is challenging. Therefore, the initiation/reintroduction of the SGLT2 inhibitor after surgery should be cautious in patients with diabetes with monitoring of clinical symptoms. If ketoacidosis is suspected, the diagnosis is made with blood gas analysis and measuring ketones in urine or blood, because glycaemia can be normal due to glucosuria. Cases of ketoacidosis in patients without known diabetes have been described.^96^ Therefore, in case of prolonged fasting, infections, or severe illness, it seems reasonable to postpone the introduction of these medications.^97^ If the oral intake is normal and no severe illness is identified, the SGLT2 inhibitor could be reintroduced 1 or 2 days after surgery.^98^

In order to prevent rehospitalization, it is important to ensure that the patient is euvolemic before discharge, as the presence of congestion is a strong predictor of readmission in patients with primary AHF.^99^ Initiation and/or optimization of guideline-directed optimal medical treatment before discharge is also key to prevent readmission.^3^ Given that a significant number of deaths occur early after discharge, the early post-operative period represents a window of opportunity to improve long-term prognosis. However, the optimal management of these patients during and immediately after hospitalization needs to be defined. Patient education regarding HF and scheduling a follow-up visit in 2–4 weeks after discharge may help further prevent new hospitalizations as well as improve prognosis. In cases of a concomitant cTn elevation, an ambulatory non-invasive functional test to detect myocardial ischaemia should be considered.

## Knowledge gaps

- The true incidence of pre-operative HF, as well as AHF, after non-cardiac surgery in the overall population is yet to be determined.
- Detailed clinical characterization of patients with chronic HF undergoing non-cardiac surgery is lacking.
- Risk factors for the occurrence of AHF after non-cardiac surgery have to be confirmed.
- Performance of the currently used pre-operative risk scores for risk prediction in patients with chronic HF is unknown.
- Andersson's score for estimation of mortality in patients with HF need external validation.
- Ideal timing for elective surgery in patients with newly diagnosed HF after introduction and optimization of HF therapy is unknown.
- Further studies are needed to determine which patients benefit from pre-operative NPs measurements.
- Exact timing for suspension and reintroduction of SGLT2 inhibitors is unclear.
- The NP cut-offs for AHF diagnosis after non-cardiac surgery are currently unclear.
- The role of surveillance with post-operative NP measurements is unknown.
- The utility of clinical decision support (e.g. through electronic medical records) for the early detection of patients at high risk for AHF after non-cardiac surgery and specific management recommendation should be examined.

## Conclusions

AHF after non-cardiac surgery is a neglected complication, yet it is associated with high mortality. There is a major need to increase awareness of the risk associated with AHF after non-cardiac surgery among several specialists including surgeons, anaesthesiologists, intensivists, internal medicine physicians, and cardiologists.

In patients with known HF, a detailed cardiac history including assessment of the NYHA functional class, clinical examination, optimization of medical treatment according to current guidelines, and ensuring euvolemia before surgery are crucial for preventing post-operative complications and reducing mortality.

For patients without known HF but with risk factors for HF, a thorough pre-operative evaluation to diagnose potential undetected HF and optimize the treatment of cardiac comorbidities is essential to reduce the likelihood of AHF after non-cardiac surgery.

Understanding the mechanisms of AHF after non-cardiac surgery is vital for prevention, which involves closely monitoring fluid status to avoid both volume overload and hypovolemia, avoid hypo- or hypertension, treating pain and anaemia to prevent tachycardia, and preventing electrolyte disturbances to avoid arrhythmias.

Vigilant post-operative care is essential for the early diagnosis and treatment of AHF after non-cardiac surgery, which could help mitigate risk and improve outcomes for patients. Cardiac biomarkers such as cTn and NPs enhance risk stratification and serve as important diagnostic tools in the perioperative setting.

Continued research and refinement of perioperative strategies are needed to further improve the care of this vulnerable patient population.

## Declarations

### Data Availability

No data were generated or analysed for this manuscript.

## Appendix Practical tips, including examples of clinical scenarios

We prepared some common scenarios of perioperative cases, as examples to help clinicians put the scientific evidence in clinical practice.

### Scenario 1: HFrEF undergoing high-risk non-cardiac surgery

This case involves a 55-year-old male patient scheduled for an elective pancreaticoduodenectomy due to pancreatic adenocarcinoma. His medical history includes arterial hypertension and chronic HF with HFrEF. The patient experiences dyspnoea consistent with NYHA Class II, with a metabolic equivalent score of over 4. The patient's vital signs include a blood pressure of 100/60 mmHg and a heart rate of 60 beats/min. His body mass index (BMI) is 22 kg/m^2^. There are no signs of congestion upon clinical examination. His medication regimen includes enalapril 10 mg twice daily, carvedilol 25 mg twice daily, spironolactone 25 mg once daily, dapagliflozin 10 mg once daily, and furosemide 40 mg once daily. Complementary examinations reveal a sinus rhythm and LV hypertrophy on the ECG. Echocardiography shows a dilated LV with an LVEF of 35% and diffuse hypokinesis. Previous coronary angiography indicates normal coronary arteries. Additionally, laboratory tests show a BNP concentration of 210 ng/L, high-sensitivity cardiac troponin T (hs-cTnT) of 16 ng/L, creatinine levels of 1.0 mg/dL, and a haemoglobin level of 130 g/L.

Comments: Although the patient has a history of HFrEF, he is currently stable, without clinical signs of congestion, and under optimal HF medical treatment. Recent assessment of LVEF showed reduced LVEF, whereas the BNP concentrations were acceptable. According to the Revised Cardiac Risk Index, the risk of post-operative complications is 10% (Class III), and according to the Andersson's score, the mortality risk is 2%–5%. Considering that the surgery is time-sensitive and curative for the underlying disease, this patient should be referred for surgery as soon as possible. It is advised to continue current HF medications (except the SGLT2 inhibitor, which should be stopped 3 days before the procedure) until surgery.^50^ Close monitoring of fluid balance throughout the perioperative period is necessary, with resumption of medications as soon as possible after surgery. Monitoring of electrolytes, renal function, hs-cTn, haemoglobin, and thrombosis prophylaxis with low-molecular-weight heparin is also advised.

Follow-up: The patient underwent surgery without complications and was safely discharged. Given the possibility of late episodes of AHF after non-cardiac surgery, an outpatient cardiology consultation for clinical evaluation in 2 weeks was scheduled.

### Scenario 2: post-operative cardiac consultation in a patient without known HF undergoing urgent moderate-risk surgery

This case involves a 74-year-old female patient admitted to the orthopaedic surgery department due to a right knee joint dislocation, rendering her unable to walk. Her medical history includes arterial hypertension, Type 2 diabetes mellitus managed with insulin, COPD, and obesity with a BMI of 41.8 kg/m^2^. The ECG showed a sinus rhythm without signs of ischaemia or arrhythmias. The knee operation was performed without problems, and she was transferred to the orthopaedic ward. On the second post-operative day, a cardiac consultation was performed due to a PMI detected during screening with hs-cTnT (pre-operative hs-cTnT was 28 ng/L, 33 ng/L on the first post-operative day, and 47 ng/L on the second post-operative day, e.g. Delta 19 ng/L). The patient complained of orthopnoea since the previous day but no chest pain. She reported dyspnoea (NYHA Class II) for 2 years, attributed to obesity and sedentarism. Clinical examination revealed pulmonary rales, jugular vein distension, and peripheral oedema. Blood pressure was 160/90 mmHg, and heart rate was 102 beats/min. The ECG showed sinus rhythm without ischaemic changes. The NT-proBNP was 3156 ng/L. Haemoglobin concentrations were 100 g/dL, and creatinine was 1.3 mg/dL. Echocardiography showed enlarged atria, concentric remodelling of the LV, and preserved LVEF. Pre-operative NT-proBNP concentrations were 594 ng/L. The patient was receiving intravenous fluids, and antihypertensive medications were discontinued on the day of the operation.

Comments: This patient experienced an episode of AHF after non-cardiac surgery due to HFpEF that was diagnosed because of a cardiac consultation triggered by perioperative troponin screening. Around 4% of PMI cases are due to AHF after non-cardiac surgery, and these patients have high rates of mortality (49%) and major adverse cardiac events (56%) within 1 year.^100^ Likely causes for the episode include a combination of volume overload, hypertension, anaemia, and tachycardia due to post-operative inflammation. Differential diagnosis with pulmonary embolism should be considered; however, the patient was under recommended prophylaxis, had no hypoxemia, no signs of deep venous thrombosis, and clinical/echocardiographic features suggested pulmonary congestion and left heart decompensation. It is also probable that this patient had undiagnosed chronic HF before the operation, indicated by dyspnoea (NYHA Class II), elevated NT-proBNP above diagnostic thresholds for HF (even considering underestimation due to obesity), and compatible structural heart abnormalities. The pre-operative NT-proBNP was already elevated, indicating a higher risk for post-operative complications and mortality. Even before measuring the NPs in the chronical setting, an HF diagnosis should have been suspected; once she had, for example, a H2FPEF score^101^ of at least 5 points, which confers a probability of HF of 80%. Given the urgent nature of the operation, there was no time for additional testing or therapies before surgery, but closer monitoring of volume status and early symptoms or signs of decompensation were warranted. If it had been an elective surgery, performing an echocardiogram before surgery and optimizing medical treatment of comorbidities and volume status could have prevented AHF after non-cardiac surgery.

Follow-up: Intravenous fluids were stopped, and intravenous loop diuretic, MRA, and SGLT2 inhibitor were initiated, with the antihypertensive treatment resumed. Due to multiple risk factors for coronary artery disease (CAD) and PMI, chronic CAD could not be excluded. Statins were started, and the patient was referred for a non-invasive ischaemic test after discharge. Early follow-up ambulatory consultation around 2 weeks post-discharge is advised to reduce the risk of readmission due to new decompensation episodes.

### Scenario 3: patient without known HF undergoing urgent moderate-risk non-cardiac surgery

This case involves an 83-year-old male patient admitted emergently to the orthopaedic surgery department due to a right pertrochanteric femur fracture caused by a non-syncopal fall. His medical history includes arterial hypertension managed with valsartan. He had no cardiovascular complaints. While no recent ECG is available, a transthoracic echocardiogram (TTE) performed 6 months prior to the operation showed an LVEF of 67% with no wall motion abnormalities. The TTE also revealed Grade II diastolic dysfunction, a systolic pulmonary artery pressure of 41 mmHg, and mild aortic stenosis. The patient underwent an open reduction and internal fixation for the right femur fracture. The patient's perioperative and post-operative course was uncomplicated. The perioperative troponin screening did not indicate a PMI but revealed a chronic high-sensitivity troponin elevation without dynamics (hs-cTnT 24 ng/L), thus not warranting a cardiology consultation. Post-operative pain was well-controlled. Clinical examination showed a dry wound dressing with intact peripheral circulation, motor function, and sensation in the right lower extremity. Mobilization proceeded without issues, and he was transferred to a rehabilitation institution. On the 15th post-operative day, the patient was referred back to the hospital due to progressive dyspnoea and was diagnosed with AHF.

Comments: Although approximately half of AHF after non-cardiac surgery cases occur in the first week after surgery, the risk persists in the first 30 days, being higher in the first 15 days post-surgery. Patients undergoing orthopaedic surgery and urgent or emergency surgery are particularly at high risk for AHF after non-cardiac surgery. Although only 20% of acute HF after non-cardiac surgery patients meet the criterion for PMI, about 80% have chronic myocardial injury, i.e. pre-operative hs-cTn levels above the 99th percentile of the URL. These patients require close monitoring for signs of acute HF after non-cardiac surgery. Retrospectively, this patient had NT-proBNP concentrations before discharge of 2621 ng/L. It is well known that, in patients discharged after an episode of primary AHF, the presence of congestion signs and elevated NP concentrations at discharge are strong predictors of readmissions due to AHF.^99,102^ Therefore, early diagnosis and treatment of congestion are essential.

Follow-up: intravenous loop diuretic, MRA, and SGLT2 inhibitor were initiated, an echocardiography was performed, which revealed stable findings compared with the pre-operative echocardiography, and the patient could be safely discharged after some days, with a recommendation of a cardiac consultation for clinical assessment in 2–4 weeks.
